# The phonon quantum of thermal conductance: Are simulations and measurements estimating the same quantity?

**DOI:** 10.1126/sciadv.adi7439

**Published:** 2023-10-13

**Authors:** Carlos A. Polanco, Ambroise van Roekeghem, Boris Brisuda, Laurent Saminadayar, Olivier Bourgeois, Natalio Mingo

**Affiliations:** ^1^ Université Grenoble Alpes, CEA, LITEN, 17 rue des Martyrs, Grenoble, France.; ^2^ Université Grenoble Alpes, CNRS, Grenoble INP, Institut Néel, Grenoble, France.

## Abstract

The thermal conductance quantum is a fundamental quantity in quantum transport theory. However, two decades after its first reported measurements and calculations for phonons in suspended nanostructures, reconciling experiments and theory remains elusive. Our massively parallel calculations of phonon transport in micrometer-sized three-dimensional structures suggest that part of the disagreement between theory and experiment stems from the inadequacy of macroscopic concepts to analyze the data. The computed local temperature distribution in the wave ballistic nonequilibrium regime shows that the spatial placement and dimensions of thermometers, heaters, and supporting microbeams in the suspended structures can noticeably affect the thermal conductance’s measured values. In addition, diffusive transport assumptions made in the data analysis may result in measured values that considerably differ from the actual thermal conductance of the structure. These results urge for experimental validation of the suitability of diffusive transport assumptions in measuring devices operating at sub-kelvin temperatures.

## Teaser

Simulations of measuring platforms for the phonon thermal conductance quantum reveal the importance of phonon wave-like effects.

## INTRODUCTION

The quantum of thermal conductance (QTC) is one of the fundamental transport quantities of meso- and nanoscopic physics, as it sets a limit to the amount of heat, entropy, and information that can flow through an individual quantum channel (*1*, *2*). This quantity describes the ratio of maximum heat flowing through a quantum channel, over the temperature difference between the thermal reservoirs driving this heat. After its theoretical prediction in the early 1980s, the QTC has been robustly measured by independent groups in systems where electrons (*3*–*5*) and photons (*6*, *7*) are the main heat carriers. However, measuring the phonon-mediated QTC has been a more elusive endeavor.

**Fig. 1.**
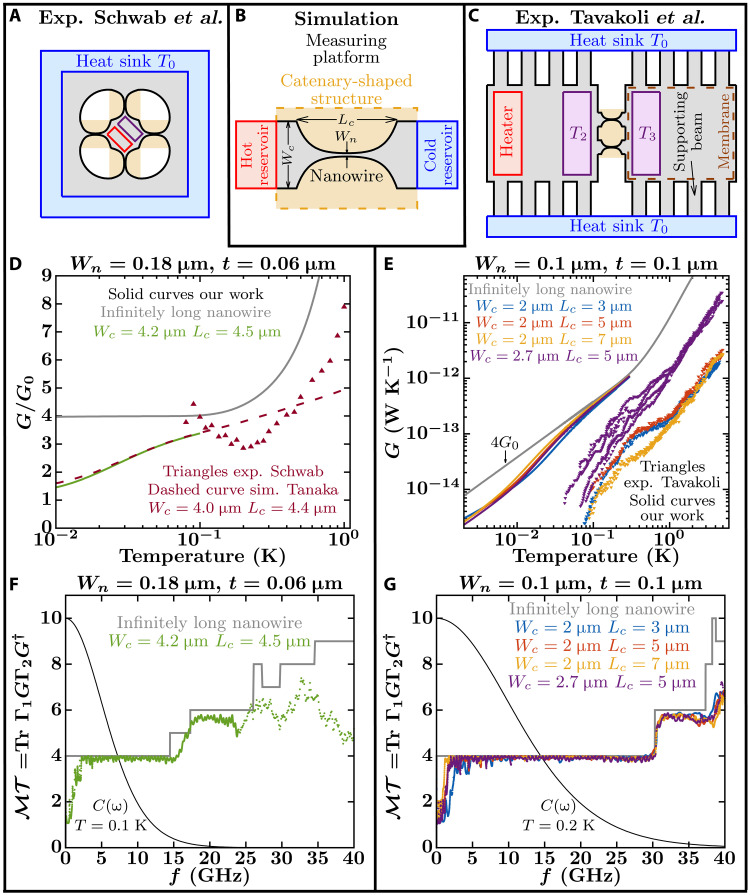
Comparison of experimental and simulated thermal conductance. (**A**) Simplified sketch of the top view of the experiment in (*9*). (**B**) Simplified sketch of a catenary-shaped structure connected to ideal thermal baths that drive heat across the nanowire. (**C**) Simplified sketch of the top view of the experiment in (*14*). (A) to (C) The catenary-shaped structure of interest is highlighted by a yellowish-shaded area. Outside this yellowish region is the measuring platform with the heat sources (heaters) represented by reddish regions, the thermometers by purple regions, and the heat sinks by blue regions. The suspended SiN sheets correspond to the gray-shaded regions. (**D**) Thermal conductance normalized by the QTC, $G_{0}=(\pi^{2}k_{B}^{2}T)/(3h)$. Solid curves show our NEGF simulations for an infinitely long nanowire with a cross-sectional area of 180 nm by 60 nm (gray curve) and for a catenary-shaped structure (green curve) similar to that in Schwab’s experiment (*9*). Reddish triangles correspond to measurements in (*9*), and the dashed reddish curve shows previous calculations in (*10*). (**E**) NEGF calculations of thermal conductance for an infinitely long nanowire with a cross-sectional area of 100 nm by 100 nm (gray curve) and for catenary-shaped structures (blue, red, yellow, and purple curves) similar to those in Tavakoli’s experiments (*14*). Triangles show measurements in (*14*). (**F** and **G**) Total phonon transmission computed from NEGF for the nanowires and catenary-shaped structures of subfigure (D) and (E), respectively. The monotonically decreasing black curves correspond to the mode heat capacity (see Eq. 1) at the labeled temperatures normalized by 10/*k*_B_.

In 1998, a seminal article theoretically showed how the shape of a nanoconstriction affects its ability to transmit phonons and suggested a catenary-shaped constriction [see Fig. 1 (A to C)] to improve the chances of observing quantized thermal conduction by individual phonon channels (*8*). Following this idea, Schwab *et al.* (*9*) carried out an experimental measurement of quantized thermal conductance (see Fig. 1A), in apparently good agreement with the earlier theoretical predictions for a single scalar field quantum channel in the catenary-shaped structure. Subsequent attempts to model the structure beyond the single-channel scalar field case, however, unveiled discrepancies between predicted and measured data (*10*).

Although more theoretical developments, including phonon nonequilibrium Green functions (NEGFs), have known important progress since then (*11*), the problem of measuring the phonon QTC in suspended microstructures has not been very much addressed from the theoretical point of view after 2005. Computing power at the time was not enough to tackle the micrometer-sized experimental structures without using simplifications. Only recently, an implementation of NEGF with finite elements has been used to simulate phonon transport in several model systems (*12*). This represents a step forward with respect to previous models, as it investigates vector (rather than scalar) phonon fields in fully three-dimensional (3D) realistic structures. Despite this capability, no theoretical investigation has yet directly tackled the measurement problem. Experimentally, there has been further reporting on the direct detection of the phonon QTC (*13*). However, recent attempts at a more detailed measurement using a setup different from Schwab *et al.*’s (see Fig. 1C) have been inconclusive regarding whether the phonon QTC can be easily observed (*14*). This poses the question of whether the difficulties to experimentally detect the theoretically predicted quantized conductance per phonon channel may result from some parasitic effects at the material or interface level or perhaps stem from the measurement design or the analysis assumptions.

To address this question, we have carried out massively parallel calculations of phonon conduction in suspended structures similar to those in the experiments in (*14*) and (*9*) using a fully 3D implementation of NEGF for the phonon field of an elastic medium. The results unveil important aspects of experimental design, where intuitive assumptions may lead to “measured” conductance values that ostensibly disagree with the actual thermal conductance. These aspects explain part of the difficulties encountered in the quest to observe the phonon QTC and should be taken into account when designing low-temperature nanoscale devices operating in the quantum regime.

## RESULTS

### Simulated conductance of ideal geometry models compared to experimental values

To simulate lattice thermal conductance at sub-kelvin temperatures, we assume that heat propagates in a continuous medium, described as a harmonic system, i.e., neglecting inelastic phonon scattering processes arising from the anharmonic part of the potential. The calculations use the NEGF formalism (*11*) adapted to a continuous medium description using the linear theory of elasticity and finite element analysis techniques (see Materials and Methods). Our simulations capture coherent phonon transport in 3D geometrical structures, including confinement effects and elastic phonon scattering from surfaces, boundaries, and material interfaces.

Within the NEGF formalism, the phonon thermal conductance can be expressed similarly to Landauer’s formulation as (*11*, *15*)

$$G=\int_{0}^{\infty }\frac{d\omega }{2\pi }C(\omega )MT(\omega )$$ (1)

with ω being the angular frequency, $C(\omega )=\hbar \omega \frac{\partial N}{\partial T}$ being the mode heat capacity, ℏ being the reduced Planck constant, *N* being the Bose-Einstein distribution, *T* being the temperature, and ℳ𝒯(ω) being the sum of transmissions across the region of interest from all the phonon modes in the hot reservoir to all the phonon modes in the cold one (see Materials and Methods). In particular, for a system with a single quantum transport channel with perfect phonon transmission [ℳ𝒯(ω) = 1], Eq. 1 yields the QTC, $G_{0}=(\pi^{2}k_{B}^{2}T)/(3h)$.

#### Expectations from early calculations

Early calculations for infinitely long dielectric nanowires showed that the QTC should be observable only up to temperatures below the temperature equivalent energy of the lowest optical phonon subbands (*8*). This upper bound temperature is typically less than 1 K and depends on the cross-sectional area and elastic properties of the nanowire. Above this temperature, the phonon distribution tail begins to reach the optical subbands, and the conductance switches from a linear to a cubic temperature dependence [gray curve in Fig. 1 (D and E)]. For instance, infinitely long SiN nanowires similar in cross-section to those used by Schwab *et al.* (*9*) (with a cross-sectional area of 200 nm by 60 nm) and Tavakoli *et al.* (*14*) (with a cross-sectional area of 100 nm by 100 nm) present a perfect phonon transmission over the four acoustic subbands up to ~15 and ~30 GHz, respectively, above which optical subbands become available for transport. Thus, their upper bound temperatures to observe the QTC are around 0.1 and 0.2 K, respectively (Fig. 1, F and G). Decreasing the cross-sectional area of the nanowire increases the upper bound temperature at which the QTC can be observed. For instance, SiN nanowires with cross-sectional areas of 50 nm by 50 nm and 25 nm by 25 nm have upper bound temperatures of about 0.4 and 0.8 K (see fig. S1).

To measure the QTC, a nanowire needs to be smoothly connected to the thermal reservoirs to minimize reflections that affect the unity transmission per transport channel (see Fig. 1B). A good choice is to join the nanowire to nanoribbon-shaped reservoirs with the same thickness (*t*) by smoothly changing its width (*w*) according to a catenary function (*8*)

$$w(z)=\pm \frac{W_{n}}{2}\cosh^{2}\left(\frac{z}{\lambda }\right),\lambda =L_{c}{\left[2\cosh^{-1}\left(\sqrt{\frac{W_{c}}{W_{n}}}\right)\right]}^{-1}$$ (2)

with *W_n_* and *W_c_* being the widths of the nanowire and nanoribbon, respectively, and *L_c_* and λ being the length and characteristic length of the catenary-shaped structure (see Fig. 1B). The intuitive meaning of λ is the length of the wire over which its cross section can be regarded to be uniform (*10*).

The calculated phonon transmission in SiN catenary-shaped structures closely follows that of infinitely long nanowires except at the lower frequencies [below 4 GHz in Fig. 1 (F and G)]. Increasing λ decreases this lower frequency limit (Fig. 1G) (*8*, *10*). Thus, the conductance approaches 4*G*_0_ as the temperature tends to the upper bound temperature, and the approaching rate is faster for structures with larger λ [see Fig. 1 (D and E)]. For the catenary-shaped structures simulated in Fig. 1E, the conductance reaches 3.48*G*_0_, 3.69*G*_0_, 3.79*G*_0_, and 3.64*G*_0_ at the upper bound temperature (*T* = 0.2 K) for the blue, red, yellow, and purple curves, respectively. Above 0.3 K, the conductance of the catenary-shaped structures should transition to that of a 3D system, closely following that of the infinitely long nanowires [gray curves in Fig. 1 (D and E)]. However, our available computing power prevents us from using grid sizes tighter than *l* = 0.05 μm (Fig. 1, E and G) and *l* = 0.06 μm (Fig. 1, D and F), which limits the maximum temperatures of these simulations to about 0.2 and 0.1 K, respectively (see Materials and Methods).

#### Comparison with experimental values

Our calculated thermal conductances disagree with measured data on catenary-shaped structures of the same size and shape as in (*14*) (Fig. 1E). Whereas all the calculations converge into a single curve above 0.2 K, experimental curves are spread over an order of magnitude at any temperature. In particular, simulations predict a similar conductance for systems with *W_c_* = 2.0 μm and *W_c_* = 2.7 μm, while experimental data show an order of magnitude difference between the two systems above 1 K. Regarding the experiment by Schwab *et al.* (*9*), our simulations do fit the measurements to a certain extent, yielding a similar overall value and trend (see fig. S2). However, they do not reproduce the curve’s fine detail, a problem that has remained unresolved for over 20 years. Specifically, our calculations, as well as previous ones including only the six lowest vibrational modes (*10*), show a normalized conductance that increases monotonically with temperature, opposite to the experimental trend between 0.08 and 0.2 K (Fig. 1D). Moreover, previous calculations in SiN catenary-shaped structures similar to those used in Schwab’s experiment suggested that λ > 5 μm is necessary to observe the plateau in normalized conductance that demonstrates the existence of the QTC before the contribution of optical subbands prevents its observation [see figure 5 from (*10*)]. However, existing experimental setups in (*9*, *14*) had λ < 1.6 μm.

The lack of agreement between simulations and experiments hints at potential difficulties to observe phonon quantized thermal conduction in suspended structures. Although the disagreement might perhaps indicate that elastic-coherent-wave-like vibrational lattice transport is not the dominant heat transport process in SiN catenary-shaped structures at the microscale at sub-kelvin temperatures (see Discussion), it may also be the result of experimental limitations. Regarding the latter, a measurement of the QTC ideally requires two blackbody reservoirs at different thermal equilibrium temperatures, as described by Swartz and Pohl (*16*) (Fig. 1B). However, at sub-kelvin temperatures, the phonon-phonon interaction processes that drive a phonon distribution toward equilibrium are weak. Therefore, the membranes that are intended to behave as reservoirs may not be at thermal equilibrium. This may cause two problems: (i) The suspended measuring platform, external to the catenary-shaped structure [see outside of the yellowish regions in Fig. 1 (A and C)], may affect the measurement, and/or (ii) the experimental protocol to extract the thermal conductance may not be appropriate. The “Influence of the measuring platform on the conductance values” and “Influence of experimental data analysis on the conductance values” sections below discuss these two problems by studying the effect of elements external to the catenary-shaped structure on the calculated conductance.

### Influence of the measuring platform on the conductance values

**Fig. 2.**
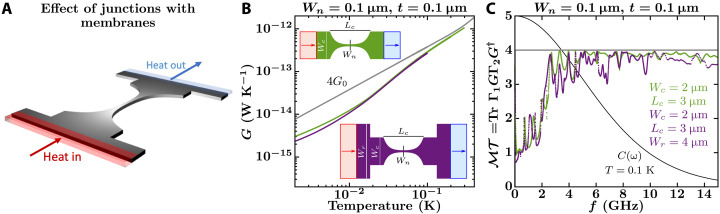
Effect on the thermal conductance of the junctions between the membranes and the catenary-shaped structure. (**A**) Sketch of a catenary-shaped structure abruptly connected to wider nanoribbons at the edges. (**B** and **C**) NEGF calculations of the conductance, *G*, and total phonon transmission, ℳ𝒯, of a catenary-shaped structure with (purple curves) and without (green curves) abrupt junctions at the edges. The structures in question are shown as insets and are defined by *W_c_* = 2 μm, *L_c_* = 3 μm, *W_n_* = 0.1 μm, *t* = 0.1 μm, and *W_r_* = 4 μm. The monotonically decreasing black curve in (C) corresponds to the mode heat capacity (see Eq. 1) at the labeled temperature normalized by 5/*k*_B_. The gray curves in (B) and (C) show the conductance and total transmission of an infinitely long nanowire with a cross-sectional area of 0.1 μm by 0.1 μm.

**Fig. 3.**
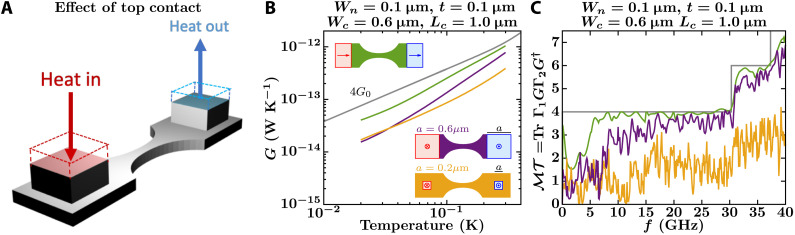
Effect on the thermal conductance of the top contacts. (**A**) Sketch of a catenary-shaped structure where heat is injected and ejected perpendicular to the plane of the structure. (**B** and **C**) NEGF calculations of the conductance, *G*, and total phonon transmission, ℳ𝒯, of a catenary-shaped structure with heat injected and ejected parallel (green curves) and perpendicular (yellow and purple curves) to the plane of the structure. For those structures with heat injected perpendicular to the structure plane, the contacts are semi-infinite squared nanowires with side *a* = 0.2 μm (yellow curves) and *a* = 0.6 μm (purple curves). The top views of the structures being compared are depicted as insets, with the catenary-shaped structures defined by *W_c_* = 0.6 μm, *L_c_* = 1 μm, *W_n_* = 0.1 μm, and *t* = 0.1 μm. The gray curves in (B) and (C) show the conductance and total transmission of an infinitely long nanowire with a cross-sectional area of 0.1 μm by 0.1 μm.

**Fig. 4.**
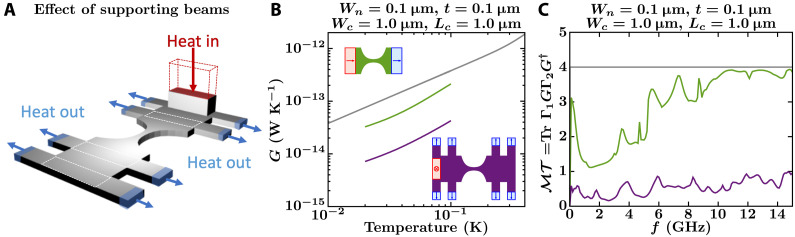
Effect on the thermal conductance of the supporting beams. (**A**) Sketch of a catenary-shaped structure with the supporting beams that hold the structure suspended. In this case, heat is injected perpendicular to the plane of the structure, and it is ejected parallel to the plane of the structure at the edges of the beams. (**B** and **C**) NEGF calculations of the conductance, *G*, and total phonon transmission, ℳ𝒯, of a catenary-shaped structure with (purple curves) and without (green curves) supporting beams. For the structure with beams, conductance and transmission are computed from the heater to all the heat sinks on the opposite side of the nanowire. Insets show the top view of the structures being contrasted, with the catenary-shaped structures defined by *W_c_* = 1 μm, *L_c_* = 1 μm, *W_n_* = 0.1 μm, and *t* = 0.1 μm. The gray curves in (B) and (C) show the conductance and total transmission of an infinitely long nanowire with a cross-sectional area of 0.1 μm by 0.1 μm.

To evaluate the first category of possible experimental problems indicated above, we consider three design features in the actual devices: abrupt width changes at the junction between the catenary-shaped structure and the membrane (Fig. 2A), thermal reservoirs that inject phonons perpendicular to the catenary-shaped structure (Fig. 3A), and the effect of supporting beams that hold the platform suspended (Fig. 4A).

#### Effect of the junctions between the catenary-shaped structure and the membranes

Adding two abrupt changes in width at the edges of the catenary-shaped structure, as done in Tavakoli’s experiment, decreases the calculated conductance only slightly (see Fig. 2B). Thus, differences between simulations and experiments cannot be explained by the presence of such abrupt junctions. Figure 2A shows a catenary-shaped structure, where the width of the nanoribbon increases abruptly from *W_c_* = 2 μm to *W_r_* = 4 μm. The total phonon transmission for such a structure (purple curve) is compared to that from a structure without the abrupt junction (green curve) in Fig. 2C. Both functions follow a similar trend except for localized resonances and antiresonances up to about 10 GHz. At higher frequencies, the mesh density (*l* = 0.1 μm) is not fine enough to describe vibrations properly. Increasing the mesh density should increase the frequency interval where the transmission functions of the two systems are similar. Therefore, we expect that the conductance of the catenary-shaped structure with abrupt junctions closely follows that of the catenary-shaped structure without abrupt junctions from 0.02 to 0.3 K and that of the infinite nanowire after 0.3 K.

#### Effect of injecting heat normal to the catenary-shaped structure plane

In experiments, heat is not coming from a semi-infinite nanoribbon-shaped contact but is injected by a heater touching the top surface of a membrane connected to the catenary-shaped structure (Fig. 3A). Part of the injected heat scatters at the bottom surface of the membrane back into the heater. Thus, the thermal conductance of the catenary-shaped structure with top contacts (insets with yellow and purple structures in Fig. 3B) can be less than that of the catenary-shaped structure with side contacts (inset with green structure in Fig. 3B). To evaluate such decrement in conductance, we calculate phonon transmission across catenary-shaped structures with top contacts of various sizes. As the contact area of the heater with the membrane increases, phonon transmission and conductance tend to that of the catenary-shaped structure with side contacts (Fig. 3, B and C). In particular, when the contact area of the heater is 1.2 μm by 1.2 μm, the phonon transmission oscillates around 3.5 at 10 GHz and its average continues to increase for larger frequencies (see fig. S3), pushing the conductance closer to four times the QTC. Because, in both experiments, the contact area of the heater with the membrane is larger than 2 μm by 2 μm, the top-grown monolithic junction cannot account for the differences between simulations and experiments in Fig. 1. Nevertheless, a very rough or patchy contact between the heater and the platform might result in a smaller contact area than the apparent one. Experiments varying the contact size might be able to check for any such contact effects.

#### Effect of beams that keep the catenary-shaped structure suspended

In the experiment by Tavakoli *et al.* (*14*), the suspended membranes plus catenary-shaped structures are supported by 2-μm-wide beams (Fig. 4A). These beams influence the vibrational modes of the systems and the phonon transmission across the nanowire. Figure 4 (B and C) shows the partial conductance and phonon transmission function from the heater to the heat sinks on the beams at the opposite side of the nanowire (see Materials and Methods). The transmission across the nanowire is strongly suppressed (≪4), which limits the observation of the QTC. As we explain in the next section, the experimental analysis protocol is meant to account for this leakage through the supporting beams and to single out the conductance inherent to the catenary-shaped structure itself. However, classical heat conduction assumptions do not necessarily hold in the quantum regime, which could render such analysis protocol nontrivial.

### Influence of experimental data analysis on the conductance values

In addition to the aforementioned differences between the ideal models and the actual measurement setup, a second potential source of disagreement between theory and experiment may come from the data analysis protocols. Experiments interpret measurement results via a thermal resistors model, but this may not properly account for the wave nature of lattice vibrations carrying heat and/or for the highly out-of-equilibrium nature of the phonon distribution in these systems. In this section, we discuss possible errors that may arise from implicit assumptions about the measuring platform, notably from presuming that the membranes behave like phonon “blackbodies” at thermal equilibrium. To test this, we apply the same protocol used to extract the experimental conductance to our phonon transport simulations, in which lattice vibrations behave as elastic and coherent waves.

#### Following the protocol used in the structure with supporting beams

**Fig. 5.**
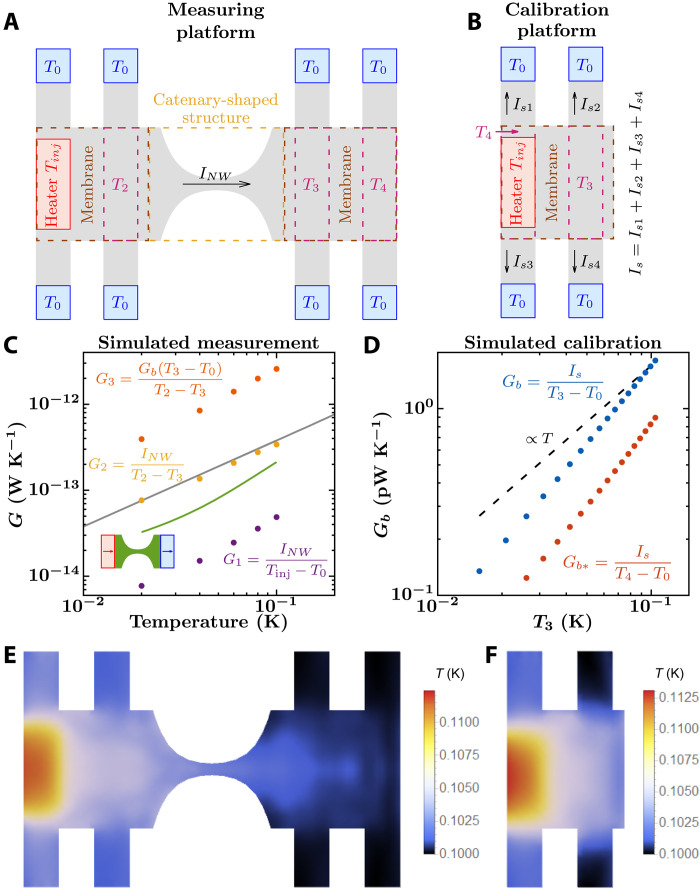
Simulation of Tavakoli’s platform to measure the thermal conductance of a nanowire. (**A** and **B**) Top view of simulated measuring and calibration platforms. Heat is injected perpendicular to the structure plane but ejected parallel to that plane, similar to Fig. 4A. The catenary-shaped structure has dimensions *W_c_* = 1 μm, *L_c_* = 1 μm, *W_n_* = 0.1 μm, and *t* = 0.1 μm, while each membrane is 1 μm by 1 μm, and the width of each supporting beam is 0.3 μm. The thermometer labeled *T*_4_ in (B) is below the heater. In addition, note that the heater is above the plane of the structure, while all the thermometers are in the top surface plane of the structure. (**C**) Simulated conductance measurements of the catenary-shaped structure within the measuring platform (purple, yellow, and orange dots). The green curve shows the conductance of the catenary-shaped structure with ideal heat baths at its edges, as shown by the inset, and the gray curve shows the conductance of an infinitely long nanowire with a cross section of 0.1 μm by 0.1 μm. (**D**) Simulated conductance measurements of the supporting beams (*G_b_* and *G*_b__*_) within the calibration platform. Conductance is computed from the heater to all the heat sinks. (**E** to **F**) Computed temperature profiles at the top plane of the measuring and calibration platforms when the heater and chamber temperatures are set to *T*_inj_ = 120 mK and *T*_0_ = 100 mK, respectively.

Let us evaluate the conductance of a catenary-shaped nanowire mimicking Tavakoli’s experiment (*14*), via different protocols. For this, we consider a catenary-shaped structure embedded in the measuring platform shown in Fig. 5A, which includes two membranes, eight supporting beams, a heater, and three thermometers. The heater is modeled as a semi-infinite nanowire perpendicular to the top surface of the platform acting as a thermal reservoir (as in Fig. 4A). The thermometers are assumed to be ideal, so they sample the average local temperature of the surfaces without perturbing them. The local temperature in nonequilibrium is the temperature that an infinitesimally small local thermometer would measure. Thus, local temperature relates to local energy content, and it is defined as the temperature that yields the same calculated energy content when a Bose-Einstein distribution is assumed (see Materials and Methods). For each chamber temperature *T*_0_, we set the heater temperature to *T*_inj_ = 1.2*T*_0_ and calculate the current flowing through the nanowire, *I*_NW_, as well as the temperatures on the thermometers—*T*_2_, *T*_3_, and *T*_4_—using the Green’s function approach. With these values, one could define the conductance of the nanowire in several ways

$$G_{1}=\frac{I_{\mathrm{NW}}}{T_{\mathrm{inj}}-T_{0}},G_{2}=\frac{I_{\mathrm{NW}}}{T_{2}-T_{3}},G_{3}=\frac{G_{b}(T_{3}-T_{0})}{T_{2}-T_{3}}$$ (3)

*G*_1_ defines a conductance between the ideal thermal reservoirs of the calculation, placed out of the system and defined to be at thermal equilibrium. This would be a “theorist’s” thermal conductance of the nanowire embedded in the measuring platform, where the role of blackbodies, as defined by Swartz and Pohl (*16*), would be played by the heater and the chamber. *G*_2_ defines a conductance of the nanowire assuming that the regions close to the catenary-shaped structure acting as thermometers are at thermal equilibrium and using the direct knowledge of *I*_NW_, which is available in some experiments (*9*) but only indirectly in others (*14*). *G*_3_ is the conductance obtained using Tavakoli’s protocol: in addition to assuming that *T*_2_ and *T*_3_ represent equilibrium “blackbody” temperatures, *G*_3_ also estimates *I*_NW_ using the conductance of the beams *G_b_*, which is measured from the calibration platform shown in Fig. 5B.

In the idealized, infinitely long side contact nanoribbon structure of the early calculations (Fig. 1B), the measurement would yield the theoretical conductance of the catenary-shaped structure (green curve in Fig. 5C). *G*_1_ lies below these values because the heat current injected at the heater can leak through the supporting beams before reaching the catenary-shaped nanowire (purple dots in Fig. 5C). Thus, for the temperature difference *T*_inj_ − *T*_0_, the current crossing the nanowire is less than that without supporting beams. In addition, injected phonons incoming perpendicular to the top surface of the platform can be reflected back into the contact at the bottom surface of the platform, lowering transmission through the nanowire.

*G*_2_, in turn, can overestimate the conductance of the catenary-shaped structure (yellow dots in Fig. 5C). This is because, as the two thermometers get closer to the nanowire, the temperature difference between them, *T*_2_ − *T*_3_, decreases below the actual temperature difference between the left- and right-traveling phonon distributions in the nanowire. If one places the thermometers directly on the nanowire and sufficiently far from its edges, then their temperatures become virtually identical (although they are located away from each other) because the ballistic nonequilibrium phonon distribution in the wire varies very little along its central segment. Thus, in this case, the measured *G*_2_ would tend to infinity. In practice, *G*_2_ could be the right way of evaluating the nanowire’s thermal conductance, provided that the two membranes behave as effective phonon blackbodies at mostly uniform temperatures and that the thermometer in the hot (cold) membrane is placed shaded from the cold (hot) phonons originated in the other membrane, which emerge out of the nanowire.

To calculate *G*_3_, *G_b_* needs to be computed first. For that, we simulate a calibration platform with four supporting beams, a heater, and two thermometers (Fig. 5B). As before, for each chamber temperature *T*_0_, the heater temperature is set to *T*_inj_ = 1.2*T*_0_ and the ensemble of the currents from the heater to all supporting beams *I_s_*, as well as the temperature at the thermometers, *T*_3_ and *T*_4_ (below the heater), are calculated. Then, the conductance of the beams, between the membrane and the surrounding material, is computed as (see Fig. 5B)

$$G_{b}=\frac{I_{s}}{T_{3}-T_{0}}$$ (4)

Figure 5D shows that *G_b_* grows faster than *T* because of the increase of total transmission with frequency from the heater to the beams (see fig. S4). Using the experimental protocol to extract *G*_3_ from the temperatures in the simulation yields conductances above the QTC (orange dots in Fig. 5C). Additionally to the error in *G*_2_, *G*_3_ also overestimates the current flowing through the beams because of the nonuniform temperature profile of the membrane: In the calibration step, *G_b_* is defined using a temperature profile where *T*_4_ > *T*_3_ (Fig. 5F), while in the actual measurement, *T*_4_ < *T*_3_ (Fig. 5E). Having the current estimated from *G_b_*(*T*_3_ − *T*_0_) is only possible if the region labeled *T*_4_ is at a higher temperature than that labeled *T*_3_, which is not the case in the temperature profile of the measurement (Fig. 5E). For instance, defining *G_b_* in terms of *T*_4_ instead of *T*_3_ as *G*_b*_ = *I_s_*/(*T*_4_ − *T*_0_) results in about half the conductance (Fig. 5D), which would halve *G*_3_ in Fig. 5. Thus, *G*_3_ overestimates the conductance of the catenary-shaped structure even more so than does *G*_2_. In contrast, measurements in (*14*) are much below theoretical expectations. We discuss possible reasons in Discussion.

Overall, the simulated local temperature profiles vary noticeably throughout the two membranes (Fig. 5, E and F). Thus, we are not in the ideal situation of two connected blackbody reservoirs proposed by Swartz and Pohl in their definition of interface thermal conductance (*16*), which challenges experimental assumptions in the data analysis protocol.

#### Following the protocol used in the beamless structure

**Fig. 6.**
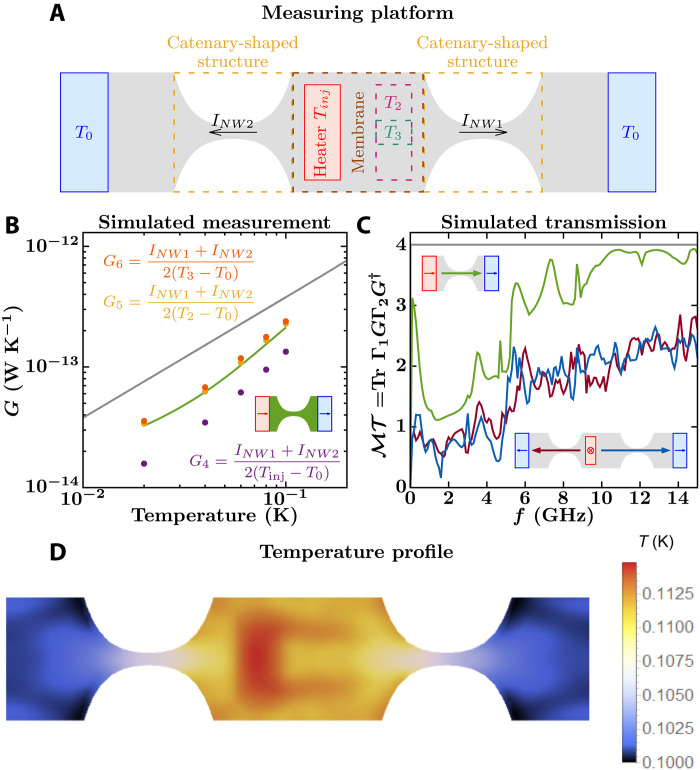
Simulation of Schwab’s platform to measure thermal conductance of a nanowire. (**A**) Top view of a simplified model of Schwab’s measuring platform. The heater injects energy perpendicular to the structure plane while the heat sinks draw energy parallel to that plane, like in Fig. 4A. The catenary-shaped structure is defined by *W_c_* = 1 μm, *L_c_* = 1 μm, *W_n_* = 0.1 μm, and *t* = 0.1 μm, and the membrane is 1.1 μm by 1 μm. (**B**) Simulated measurements of thermal conductance of the catenary-shaped structure within the measuring platform (purple, yellow, and orange dots). The conductance of an infinitely long nanowire with a cross-section of 0.1 μm by 0.1 μm is given by the gray curve and that of the catenary-shaped structure with ideal heat baths, as shown in the inset, is given by the green curve. (**C**) Total phonon transmission for the infinitely long nanowire (gray curve), for the catenary-shaped structure with ideal heat baths (green curve), and for Schwab’s measuring platform. For the latter, transmission is computed from the heater to the heat sink on the left (red curve) and to that on the right (blue curve), as shown in the lower inset. (**D**) Computed temperature profile at the top plane of the measuring platforms when the heater and chamber temperatures are set to *T*_inj_ = 120 mK and *T*_0_ = 100 mK, respectively.

We model a beamless structure, as is the case in Schwab’s experiment (*9*) sketched in Fig. 1A, as two catenary-shaped structures supporting a suspended central membrane. Heat is injected by a monolithic top-contact similar to that in Fig. 4A (heater, *T*_inj_), and temperature is measured by an ideal thermometer on the region labeled *T*_2_ (Fig. 6A). Within this measuring platform, the conductance of a catenary-shaped structure can be computed in various ways

$$G_{4}=\frac{I_{\mathrm{NW}1}+I_{NW2}}{2(T_{\mathrm{inj}}-T_{0})},G_{5}=\frac{I_{\mathrm{NW}1}+I_{NW2}}{2(T_{2}-T_{0})}$$ (5)

Similar to *G*_1_, *G*_4_ would be the theorist’s thermal conductance of the nanowire within the measuring platform because the thermal reservoirs are ideal, outside of the system, and at thermal equilibrium. On the other hand, *G*_5_ is the conductance obtained using Schwab’s protocol, where *T*_2_ is assumed to be a good estimate for the temperature of the phonon distribution within the membrane.

As expected, *G*_4_ is less than the conductance of the ideal catenary-shaped nanowire with infinitely long nanoribbon side contacts (purple dots versus green curve in Fig. 6B) because *G*_4_ includes additional phonon scattering at the junction between the top contact and the membrane. However, it is unexpected that *G*_5_ lies almost on top of the conductance of the ideal catenary-shaped structure (yellow dots in Fig. 6B). For the particular system in Fig. 6A, the phonon transmission, and thus the heat current from the heater to the cold reservoirs, are about half of what an ideal catenary-shaped structure would have (Fig. 6C), but *T*_2_ − *T*_0_ is also about half *T*_inj_ − *T*_0_. Thus, the additional thermal resistance of the top contact with the membrane is balanced by the temperature drop measured at *T*_2_.

The protocol to calculate *G*_5_ has some resilience to the nonuniform and nonsymmetric temperature profile in the membrane, which has a maximum and minimum temperature of 114.5 and 109.3 mK, respectively (Fig. 6D). On one hand, despite the lack of symmetry in the temperature profile (Fig. 6D), phonon transmissions from the heater through the catenary-shaped nanowires at either side of the membrane are similar (red and blue curves in Fig. 6C). Thus, our computed heat currents *I*_NW1_ and *I*_NW2_ differ by less than 5%. This surprising result may follow from the lack of phonon scattering in the membrane, causing phonon transmission to be dominated by scattering in the catenary-shaped structures, and in our particular case, also scattering from the top contact to the membrane. On the other hand, shrinking the thermometer from the region *T*_2_ to *T*_3_ results in almost the same conductance (orange dots in Fig. 6B), as the average temperatures over those regions differ by less than 1%. The resemblance between the conductance mimicking Schwab’s protocol (*G*_5_) and that from the ideal catenary-shaped structure with side contacts may not hold for other sizes of the measuring platform. As shown in Fig. 3C, the phonon transmission function changes with the size of the top contact. Thus, more simulations are required to evaluate the robustness of such finding.

### Nonclassical features on the temperature profiles

**Fig. 7.**
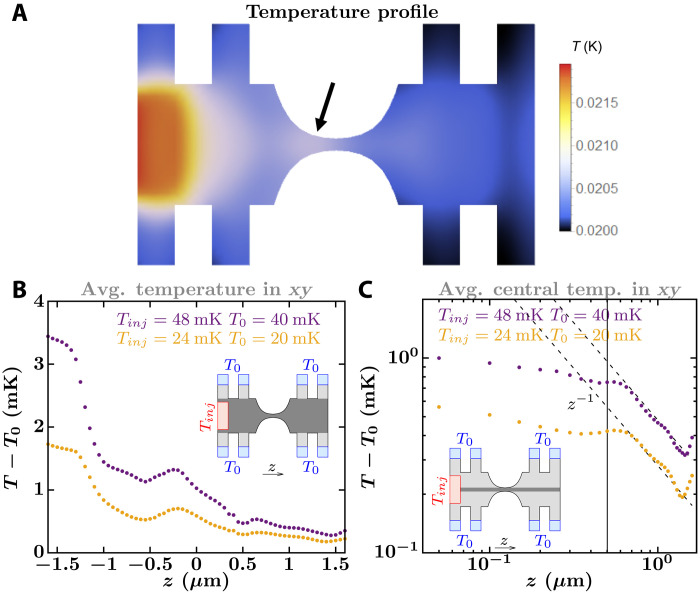
Non-Fourier features in the temperature profiles. (**A**) Simulated temperature profile at the top plane of Tavakoli’s platform to measure thermal conductance of a nanowire. The system geometry equals that of Fig. 5A, but the heater and chamber temperatures are set to *T*_inj_ = 24 mK and *T*_0_ = 20 mK, respectively. The black arrow points to a local maximum temperature. (**B**) Average temperature of the measuring platform taken in the *xy* plane but not including the supporting beams (see dark gray region in the inset). The yellow and purple curves display local maximum temperatures at about −0.25 μm. (**C**) Average temperature of the measuring platform taken in the *xy* plane over the cross-section of 0.1 μm by 0.1 μm that overlaps with the nanowire (see darker gray region in the inset). The vertical line shows the right edge of the catenary-shaped structure, and the dashed lines are proportional to *z*^−1^.

The temperature profiles of our simulations show non-Fourier features arising from wave-dominated heat transport. For example, the valley of temperature close to the right edge of the membrane on the right in Fig. 7A would imply the existence of a heat sink in the diffusive transport regime by virtue of Fourier’s heat equation. There is no sink, however. Instead, this valley arises from interference of phonon waves reflecting specularly from the platform edges. Another example of nonclassical temperature behavior is the local maximum of temperature in the catenary-shaped structure in Fig. 7A (see black arrow), which is also shown in Fig. 7B at around −0.25 μm. This maximum can be understood from wave properties of vibrations in a finite quantum barrier, which conspire to increase the local density of states close to the barrier. This translates into an accumulation of local energy, seen here as an increase in temperature. Similar temperature local maxima appear in 1D chains analogous to the catenary-shaped structure (see Fig. S5).

Heat transfer features arising from the ballistic nature of vibrational transport are also visible in our temperature profiles. Outside the colder part of the catenary-shaped structure, temperature decreases inversely proportional to distance, just like a radiative point source on a 2D system (see Fig. 7C and the Supplementary Materials). This is different from the behavior predicted by the heat equation, where temperature decreases logarithmically (see the Supplementary Materials). Note that on a purely ballistic system where phonon transmission equals one for each phonon subband, the temperature should be constant along the nanowire. This is not seen in the catenary-shaped structure because the width of the catenary shape changes continuously, generating reflections.

## DISCUSSION

Our simulation results unveil a non-negligible influence of the measuring platform on the experimental estimation of the QTC. At sub-kelvin temperatures, wave-dominated heat conduction can extend for up to tens of micrometers, permeating the sample of interest and the measuring device (*17*–*19*). Thus, a fully nonequilibrium phonon transport model is necessary not only on the sample but also on the measuring platform, including membranes, supporting beams, and heaters, to properly interpret experimental data.

However, a greater challenge when trying to measure quantized thermal conductance is for the experimental setup itself to satisfy certain criteria, as explained by Swartz and Pohl. In their article, they show that the thermal conductance between two blackbody cavities connected through a hole has a finite value, although “there is no interface except the imagined one between the two cavities, and there is no temperature *discontinuity* at that interface” (*16*). In our case too, the beginning and end boundaries of the nanowire are not well defined, and the temperature can remain rather constant along the wire, but nevertheless, its thermal conductance must have a finite value, according to Swartz and Pohl. This value is well defined only if the nanowire joins two ideal blackbody phonon reservoirs (or “cavities”), just as in the thought experiment depicted in figure 7 of (*16*). From that figure, it is also apparent that the position of the thermometers matters. Placing the thermometers just facing the nanowire on both sides would result in a vanishing temperature difference between them, yielding an infinite thermal conductance, in disagreement with Swartz and Pohl. As shown in figure 7 of (*16*), the best location for the thermometers is such that they cannot directly “see” the phonons coming out of the nanowire (which are at the other reservoir’s temperature) but only receive thermalized phonons from the reservoir that they are in. Such an ideal, uniform-temperature blackbody reservoir can however be difficult to achieve on the measuring membranes of the real experiment. If the local temperature is not uniform throughout the membranes, as our calculations suggest, this can lead to errors in the reported conductance. Furthermore, at the low temperatures and sizes involved, this variation cannot be predicted via Fourier’s heat equation but stems from ballistic and wave-mechanical phonon transport. Therefore, experimental setups must pay special attention to the topology of the measurement platforms.

The two existing topologies to measure the QTC have their own advantages and challenges. In Schwab’s topology (*9*), the supporting beams of the membrane are the nanowires themselves. Thus, the heat flowing across the nanowires equals the power dissipated in the heater, which is directly known without any approximated estimation. However, the nanowires are topped with superconducting and metal wires to drive the heater and thermometer on the membrane. Therefore, heat flowing through the top layers may influence the measurement of the nanowires’ conductance. This might be solved using alternative ways of injecting heat and measuring temperature, such as optical methods. In Tavakoli’s topology (*14*), nanowires do not have top layers, but knowing the heat flowing through them is not straightforward, as it depends on the system outside the catenary-shaped structure.

The question remains, why do our simulated results not match experimental measurements? A possible source of disagreement might be that our simulations assume coherent, elastic lattice vibrational transport in a continuum medium. Thus, they neglect three-phonon interactions, surface roughness, and the amorphous nature of SiN. At low temperatures, three-phonon scattering is generally a negligible process for lattice thermal transport (*20*, *21*). For instance, Klemens’ formula for Umklapp phonon-phonon scattering rates $\tau_{u}^{-1}=BT\omega^{2}e^{-C/T}$ in silicon (*B* = 1.73 × 10^−19^ s/K and *C* = 137.39 K) (*22*, *23*) decrease more than 60 orders of magnitude when the temperature drops from 300 to 1 K. Overall, three-phonon scattering should have negligible effects on thermal conductance below 0.1 K. Thus, those effects cannot reconcile the disagreement between simulation and experiments shown in Fig. 1.

The role of phonon scattering from rough surfaces and rough interfaces with thermometers and heaters is not included in our simulations. However, as temperature decreases, the influence of surface roughness on thermal conductivity is expected to decrease because waves with longer wavelengths are less sensitive to surface imperfections. In particular, for SiN samples like those of Tavakoli *et al.* (*14*), where the root mean square of deviation of the surface from a reference plane is about 3 nm, Ziman’s model at temperature 0.1 K predicts that most of the heat is carried by waves with surface scattering limited mean free paths larger than 12 μm (see the Supplementary Materials). Thus, most of the phonons may be negligibly affected by surface roughness. On the other hand, any defects or voids present at the interfaces of the SiN membranes with heaters and thermometers may act as diffusive scattering centers adding incoherence and destroying some of the effects discussed here that rely on coherent phonon transport. Evidence of non-negligible surface phonon scattering at sub-kelvin temperatures when submicrometer particles, as well as thin films, are deposited on SiN is found in the study of Holmes *et al.* (*24*). Further studies are necessary to evaluate the extent of this effect.

Regarding amorphous materials, their properties differ fundamentally from those in crystals. At temperatures of a few K, amorphous materials present a heat capacity larger, as well as a thermal conductivity orders of magnitude lower, than those of their crystalline counterparts (*25*, *26*). Moreover, their thermal conductivity is proportional to *T*^2^ instead of the usual *T*^3^ in crystals (*27*). These properties imply the existence of low-frequency vibrational modes and scattering processes beyond those attributed to phonons, which may be important to reconcile our simulations with experimental measurements on the QTC.

Although several models exist to explain the anomalous thermal properties of amorphous materials, there is no consensus about the microscopic mechanism causing them (*28*–*32*). Thus, here, we focus on existing experimental evidence on amorphous SiN suspended membranes at low temperatures, which is also inconclusive. On the one hand, the noncrystalline behavior of heat capacity and thermal conductivity has been demonstrated in SiN membranes with various geometries and measuring techniques (*33*–*38*), implying the existence of nonphononic lattice vibrations and scattering processes. On the other hand, ballistic and coherent thermal transport properties have also been shown in SiN membranes (*17*, *19*, *24*, *39*), implying a lack of intrinsic scattering processes and, in the coherent case, a dominant role of long-wavelength phonons in transport. It remains an open question to quantify the role of nonphononic processes in the measurement of the QTC.

Our simulations of temperature on Tavakoli’s (*14*) and Schwab’s (*9*) measuring platforms are limited to temperatures and membrane sizes about an order of magnitude smaller than those in the actual experiments. Exceeding these boundaries may blur coherent wave effects from the temperature profiles, as more phonons contribute to the heat transfer process and are involved in the spectral integrals to calculate temperatures (see Materials and Methods). Quantifying the extent of temperature changes across membranes sized as those in experiments requires further investigation. From the current state of our code, increasing the size and temperature range of our simulations requires considerable effort in code development, but besides that, the simulations are, in principle, possible.

In summary, we have simulated heat transport in existing experimental setups to measure the QTC, including the sample and the measuring platform. Our simulations assume that heat is carried by elastic and noninteracting vibrational waves, thus capturing purely wave-like heat conduction. We show that the disagreement between existing measurements and simulations on idealized catenary-shaped structures is not reconciled by including parts of the measuring platform in the simulations. Thus, wave-like phonon transport phenomena, like reflections from surfaces and junctions, along the measuring platform and sample are not the culprit for the discrepancies. Moreover, we show the detrimental effects on thermal conductance values computed with current measuring protocols that assume diffusive heat transport within the measuring platform. At sub-kelvin temperatures, wave-like heat conduction may be dominant even in the measuring platforms. Thus, future experimental efforts should account for non-Fourier heat conduction effects in parts of the measuring platform, such as suspended membranes and supporting beams. Our simulations suggest that the key to finally measuring the QTC lies in finding how to create phonon blackbody reservoirs at uniform, sub-kelvin temperatures, in quasi-2D suspended membranes.

## MATERIALS AND METHODS

We compute vibrational thermal transport properties using the NEGF formalism (*11*, *40*–*42*) adapted to work with a finite element mesh instead of atomistically (*12*). At low temperatures (~1 K), excited vibrational waves have wavelengths much larger than the atomic scale. Thus, such waves are well described by the linear theory of elasticity [see section 2.7 in (*21*)]. Discretizing the continuum displacement vector field with finite element techniques, the equation of motion in the frequency domain that dictates the dynamics of elastic waves is given by [see section 11 of (*43*)]

$$(\omega^{2}M-C)\vec{u}=\vec{0}$$ (6)

with ω being the angular frequency and $\vec{u}$ being a vector containing the deviations from equilibrium at each node. **M** is a diagonal matrix containing the masses assigned to each node *m_n_* according to the so-called lumped mass method [see section 11.3 of (*43*)], i.e., *m_n_* = ∑_β_*m*_β_/*N*_β_, with β running over the elements that contain node *n*. For each element β, *m*_β_ equals its mass and *N*_β_ is its number of nodes. **C** is the so-called element stiffness matrix containing the internode force constants, which depend on the tensor of elastic constants and the types and shapes of the elements. In particular, we use tetrahedron elements and calculate their internode force constants according to finite element techniques described in (*43*) (using equation 3.3-7, mimicking the process outlined in section 7.2 for a tetrahedron instead of a linear triangle and assembly element-wise variables according to section 2.5). The equation of motion in Eq. 6 is analogous to that describing lattice vibrations atomistically (*11*, *40*); thus, the NEGF machinery to calculate transport can be applied, replacing atoms by nodes, atomic masses by lumped masses, and interatomic force constants by internode force constants.

Within the NEGF formalism, heat is driven across the region of interest by two reservoirs set at two different thermal equilibrium temperatures. The reservoirs inject phonons with an equilibrium distribution and perfectly absorb incoming vibrations out of equilibrium. In particular, we neglect phonon-phonon interactions, as they are not important at low temperatures (~1 K); thus, thermal conductance can be expressed similar to Landauer’s formulation (*11*, *15*), as given by Eq. 1. ℳ𝒯(ω) is defined as the Trace⌈**Γ***_L_****G****^R^***Γ***_R_*(***G****^R^*)^†^⌉, with **Γ**_*L*(*R*)_ the so-called broadening matrix for the left (right) contact, ***G****^R^* the retarded Green’s function [see equations 4 to 7 in (*11*) for more details]. Trace⌈**Γ***_L_****G****^R^***Γ***_R_*(***G****^R^*)^†^⌉ equals the sum of transmissions across the region of interest from all the phonon modes in the hot reservoir to all the phonon modes in the cold one. Our choice to replace Trace⌈**Γ***_L_****G****^R^***Γ***_R_*(***G****^R^*)^†^⌉ as ℳ𝒯(ω) in Eq. 1 emphasizes the relationship between NEGF and Landauer’s formalism (*44*). ℳ refers to the number of transport channels, sub-bands available for transport or modes, and 𝒯 to an average transmission over these modes. In particular, for an infinitely long, narrow (~100 nm by 100 nm) dielectric nanowire at sub-kelvin temperatures, like the ones studied here, there are four available transport channels corresponding to one longitudinal, two transverse, and one torsional vibrational subbands, and phonon transmission is 1. Thus, the conductance of the wire equals four times the QTC (*G* = 4*G*_0_). Be aware that the retarded Green’s function is noted with a bold letter ***G****^R^*, not to be confused with conductance, which is denoted by *G*. For the calculations in Figs. 4C and 6C and fig. S4B, ℳ𝒯_*i*,*j*_(ω) = Trace⌈**Γ***_i_****G****^R^***Γ***_j_*(***G****^R^*)^†^⌉ defines the total phonon transmission from all the phonons in contact *i* to all the phonons in contact *j*, and **Γ***_i_* is the broadening matrix for contact *i*. For Fig. 4B, *G*_*i*,*j*_ is found using Eq. 1 but replacing ℳ𝒯(ω) by ℳ𝒯_*i*,*j*_(ω).

Our NEGF simulations capture energy flow carried solely by elastic lattice vibrations. Wave effects such as transmission, reflection, diffraction, and interference within the specific system geometries are fully considered. For the particular systems simulated here, SiN is considered an isotropic medium with Young’s modulus *E*_0_ = 289 GPa, Poisson’s ratio ν = 0.2, and mass density ρ = 3100 kg m^−3^ (*45*, *46*). Then, the tensor of elastic constants is defined in terms of $c=\frac{E_{0}}{\left(1+\nu )(1-2\nu \right)}$ and G= $\frac{E_{0}}{2(1+\nu )}$ as [equation 3.1-5 in (*43*)] *C*_11_ = *C*_22_ = *C*_33_ = (1 − ν)*c* = 321.1 GPa, *C*_44_ = *C*_55_ = *C*_66_ = 𝒢 = 120.4 GPa, and *C*_12_ = *C*_21_ = *C*_13_ = *C*_31_ = *C*_23_ = *C*_32_ = *νc* = 80.3 GPa. From these constants, the longitudinal and transverse sound velocities are given by $v_{L}=\sqrt{C_{11}/\rho }=10,177.63$ m s^−1^ and $v_{T}=\sqrt{C_{44}/\rho }=6232.50$ m s^−1^, respectively. The edges of the structures simulated here that are not connected to a contact are free to move.

Our in-house transport code has been benchmarked for consistency and correctness. Numerical phonon dispersions for bulk SiN yield longitudinal and transverse sound velocities *v_L_* = 10,177.60 m s^−1^ and *v_T_* = 6232.48 m s^−1^, respectively. The close agreement with the analytical predictions above validates the extraction of internode force constants from a given mesh. We also computed the phonon dispersion of GaAs nanowires, which are in excellent agreement with the analytical calculations in (*47*). Our atomistic NEGF solver has been benchmarked and used in the past to model phonon transport across GaN/AlN interfaces (*48*). The full transport code, including the finite element methods that extract node masses and internode force constants for a particular mesh integrated with the NEGF solver, was benchmarked against independent transmission and conductance calculations in defective GaAs nanowires in (*12*). Our in-house transport code is available online (*49*).

The numerical convergence of our in-house code is closely related to the mesh. The usual rule of thumb consists of choosing the maximum distance between grid points smaller than four times the minimum wavelength desired in the simulation. For example, the mesh used in the contact regions of the simulations in Fig. 1 (E and G), described by a characteristic length *l* = 0.05 μm, translates to a grid density of 64,000 nodes per μm^3^ and describes well lattice frequencies up to about 35 GHz or conductance up to 0.2 K. Most of the simulations presented here combine a periodic mesh grid at the contacts, convenient to describe infinite regions, with a randomly generated grid on the device region, convenient to easily generate meshes using existing tools. This generates two virtual thermal resistances that are not physical. The randomness of the grid acts like random impurities. Thus, larger backscattering results from larger samples and at higher frequency vibrations. At the interfaces between the ordered and disordered meshes, there is a resistance similar to the Kapitza resistance. The effect of these two virtual resistances in simulations decreases as the mesh becomes finer.

In out-of-equilibrium systems, the local temperature, as measured by an ideal thermometer, can be defined in terms of the local energy content. Intuitively, an ideal thermometer is, at every instant, taking in the phonons present in the infinitesimally small domain of space that it occupies and putting the same (infinitesimally small amount of) energy contained in those phonons back into the system but with a mode occupation distribution corresponding to that of thermal equilibrium. The local temperature at the thermometer’s position is thus the one that guarantees that the energies being taken in and out are the same. Within the Green’s function formalism, the energy taken out from node *i* by the thermometer is the local energy associated with the three degrees of freedom α of node *i*

$$E_{i}=\sum_{\alpha \in \mathrm{node}\;i}\int_{0}^{\infty }\frac{\omega d\omega }{\pi }\hbar \omega G_{\alpha }^{n}$$ (7)

with $G_{\alpha }^{n}$ being the diagonal term in row and column α of the spectral number operator defined as $G^{R}[\sum_{c}{\text{Γ}}_{c}N(T_{c})]{\left(G^{R}\right)}^{†}$, the sum running over all contacts *c*, and *N*(*T_c_*) being the Bose-Einstein distribution at the contact temperature *T_c_* [the notation follows closely that of (*11*) and that of chapters 8, 9, and 10 in (*44*); do not confuse $G_{a}^{n}$ with conductance, denoted by the unbold letter *G*]. The energy taken in by the thermometer is calculated as the local density of states weighted by an equilibrium distribution. Then, the local temperature at node *i*, *T_i_*, is the temperature for which

$$E_{i}=\sum_{\alpha \in \mathrm{node}\;i}\int_{0}^{\infty }\frac{\omega d\omega }{\pi }\hbar \omega A_{\alpha }N(T_{i})$$ (8)

with ***A***_α_ the diagonal element in row and column α of the spectral operator ***A*** = *i*[***G****^R^* − (***G****^R^*)^†^], which is related to the local phonon density of states (LDOS) at the degree of freedom α by

$${\mathrm{LDOS}}_{\alpha }=\frac{\omega }{\pi }A_{\alpha }\;\mathrm{and}\;\int_{0}^{\infty }\frac{\omega d\omega }{\pi }A_{\alpha }=1$$ (9)

In particular, Figs. 5 to 7 show an interpolation of the temperature assigned to each node.

## Supplementary Materials

### This PDF file includes:

Supplementary Text Figs. S1 to S6 References
