# Supplementary material for: The phonon quantum of thermal conductance: Are simulations and measurements estimating the same quantity?
Source: Sci Adv. 2023 Oct 13;9(41):eadi7439. doi: 10.1126/sciadv.adi7439 (PMC11090371; doi:10.1126/sciadv.adi7439)
Supplement: Supplementary file 1 — Supplementary Text Figs. S1 to S6 References [file sciadv.adi7439_sm.pdf]

Science Advances
AAAS

Supplementary Materials for

The phonon quantum of thermal conductance: Are simulations and measurements estimating the same quantity?

Carlos A. Polanco *et al.*

Corresponding author: Carlos A. Polanco, capolanco@gmail.com; Natalio Mingo, natalio.mingo@cea.fr

Sci. Adv. **9**, eadi7439 (2023)
DOI: 10.1126/sciadv.adi7439

This PDF file includes:

Supplementary Text
Figs. S1 to S6
References

Supplementary Text

<u>Phonon transmission of infinitely long nanowires</u>
Figure S1 shows the sum of phonon transmissions, $\mathcal{MT}$, for infinitely long SiN rectangular nanowires with cross-sectional area 50×50 nm^2 and 25×25 nm^2. Optical branches start at about 60 GHz and 120 GHz respectively. Thus, the quantum of thermal conductance, which is the integral of $\mathcal{MT}$ weighted by C (see Equation 1 in the main manuscript), is observable up to temperatures of about 0.4 K and 0.8 K.

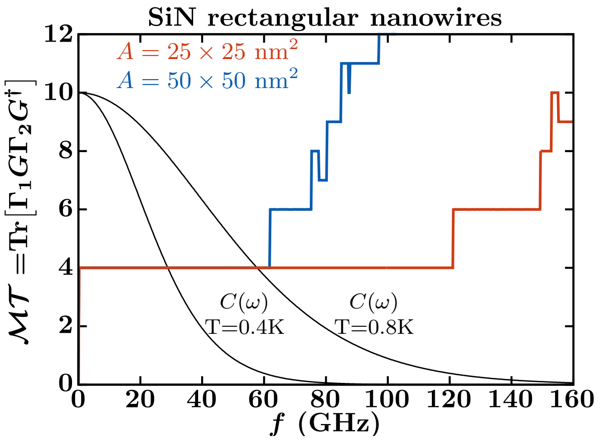

Fig. S1. Total phonon transmission for infinitely long nanowires. Curves show the sum of phonon transmissions across infinitely long, squared, SiN nanowires with cross-sectional area $\mathbf{25 \times 25}$ nm^2 (red) and $\mathbf{50 \times 50}$ nm^2 (blue). The black curves are normalized mode heat capacity at 0.4 K and 0.8 K.

Comparison of Schwab's measurements with our simulations without normalization

Figure S2 displays similarities between the thermal conductance measurements by Schwab et al. (9) (red triangles) and our simulations on infinitely long nanowires (grey solid curve). Specifically, from about 0.2 K to 1 K, simulations and measurements seem to have a similar temperature dependence. Over that temperature range, the conductance is transitioning from a linear to a cubic temperature dependence, indicating a shift in the phonon sub-bands that contribute dominantly to heat flow - from acoustic to optical sub-bands. The apparent agreement is not enough to demonstrate the existence of the QTC, which requires conductance resemblance below 0.1 K (see Fig. 1F in the main manuscript). In particular, over the temperature range below 0.2 K, the measured and simulated conductances have different temperature trends, as shown by Fig. 1D in the main manuscript.

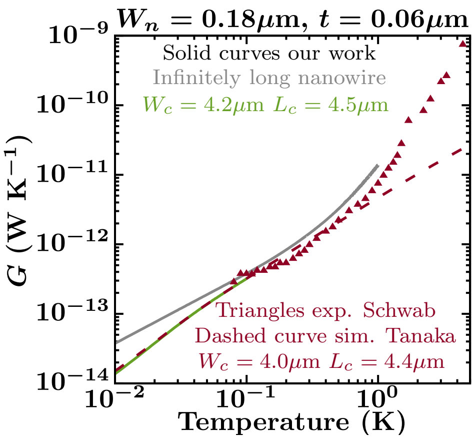

Fig. S2. Comparison of experimental and simulated thermal conductance. Thermal conductance of catenary-shaped structures similar to those measured by Schwab et al. (9). The data in this figure is the same as that shown in Figure 1D of the main manuscript. Solid curves show our NEGF simulations for an infinitely long nanowire with cross-sectional area $\mathbf{180}$ nm $\times$ $\mathbf{60}$ nm (gray curve) and for a catenary-shaped structure (green curve) similar to that in Schwab's experiment (9). Reddish triangles correspond to measurements by ref. (9) and the dashed reddish curve shows previous calculations by ref. (10).

Phonon transmission for larger top junction

Figure S3 shows the conductance and sum of phonon transmissions, $\mathcal{MT}$, for structures similar to those in Figs. 3 of the main text, but with $W_c = 1.2$ μm instead of $W_c = 0.6$ μm. As the cross-sectional area of the top junction increases, phonon transmission almost recovers its maximum value for frequencies larger than 10 GHz.

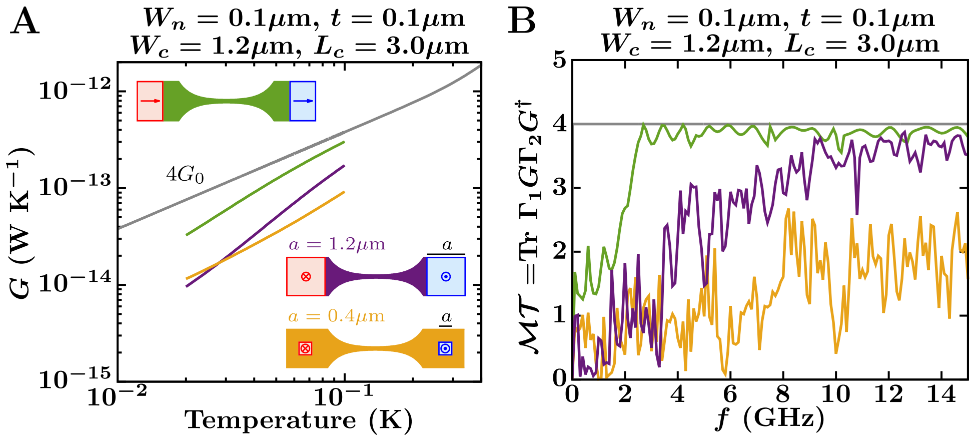

Fig. S3. Effect on the thermal conductance of the top-contacts. (**A** and **B**) NEGF calculations of the conductance, G, and total phonon transmission, $\mathcal{MT}$, of a catenary-shaped structure with heat injected and ejected parallel (green curves) and perpendicular (yellow and purple curves) to the plane of the structure. These structures are similar to those simulated in Fig. 3 of the main manuscript, but larger in size. For the structures with heat injected perpendicular to the structure plane, the contacts are semi-infinite squared nanowires with side $a = 0.4\mu$m (yellow curves) and $a = 1.2\mu$m (purple curves). The top view of the structures being compared are depicted as insets, with the catenary-shaped structures defined by $W_c = 1.2\mu$m, $L_c = 3\mu$m, $W_n = 0.1\mu$m and, $t = 0.1\mu$m.

Phonon transmission for calibration platform (Figure 5B in the main manuscript)
Figure S4B shows the sum of phonon transmissions from the heater to each supporting beam for the calibration platform shown in Figure S3A. $\mathcal{MT}$ increases with frequency as more phonon modes become available for transport in the beams. At low frequencies, $\mathcal{MT}$ is less than four due to phonon scattering from the heater to the membrane and from the membrane to the beams.

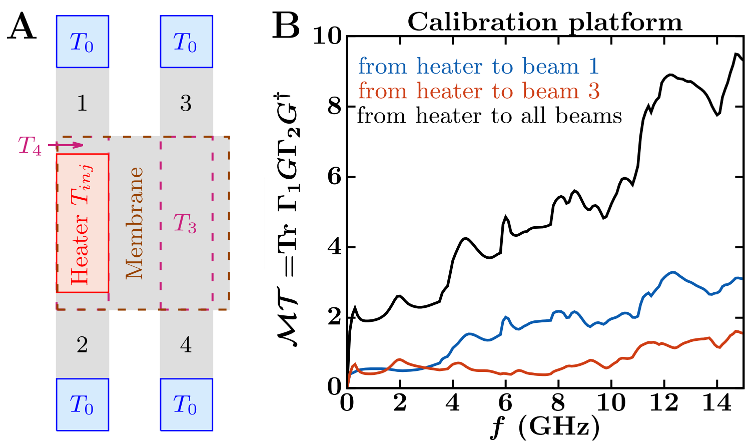

Fig. S4. Phonon transmission in Tavakoli's calibration platform. (**A**) Top view schematic of the calibration platform, including a 1μm × 1μm membrane and four beams of width 0.3μm. The thickness of the platform 0.1μm. (**B**) The sum of phonon transmissions from the heater to all beams (black curve), to beam 1 (blue curve) and, to beam 3 (red curve).

Temperature local maximum in a one-dimensional system

Consider a one-dimensional chain of atoms, where each atom has only one degree of freedom and interacts only with its nearest neighbors (Figure S5A). All interatomic force constants are set to $c = 40$ N/m, while the masses of atoms in the right and left reservoirs as well as those in atoms labeled 1-12 and 19-30 are set to $m = 4 \times 10^{-26}$ kg, and the masses of atoms 13-18 are set to $m/3$. The hot and cold reservoirs are set to 36 K and 24 K respectively. Figure S5D shows the temperature profile of such a system, displaying a local maximum between atoms 12 and 13 and a local minimum between atoms 18 and 19. These critical points in the temperature profile follow from the local density of states (LDOS) created by the sudden drop in mass, which displays a local maximum as one gets closer to the drop (Figure S5C). On the left side, as we move towards the mass-interface, the phonon distribution coming from the hot reservoir excites more states (Figure S5E), causing the increase in temperature. On the right side, as we move towards the mass-interface, the phonon distribution coming from the cold reservoir excites fewer states, because some of the states are shifting up in frequency (Figure S5E). Figure S5B shows the resonances in phonon transmission, which coincide with the quantized local density of states (Figure S5C between atoms 13-18). This resembles the states of a particle in a box, the classical problem in quantum mechanics.

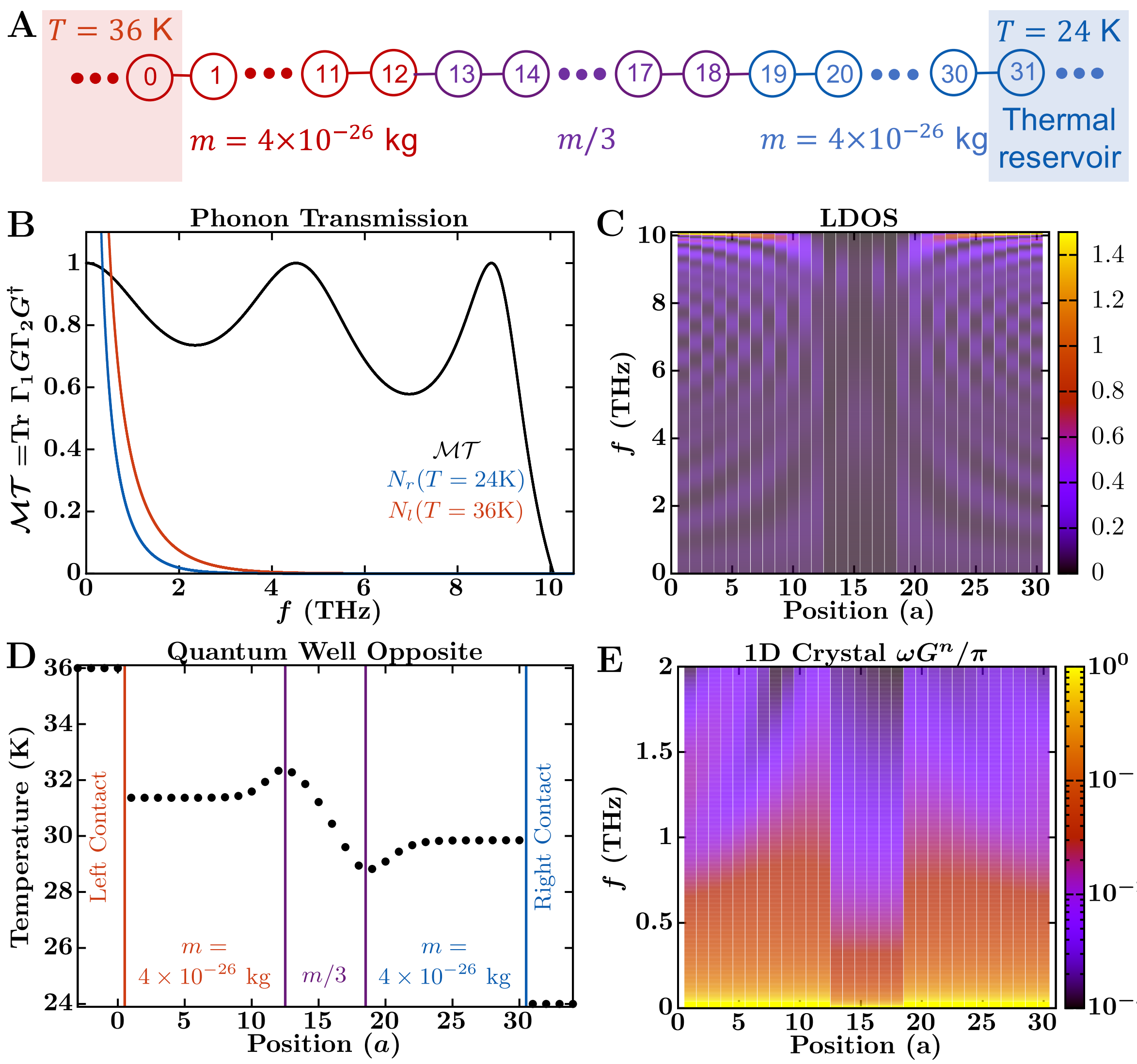

Fig. S5. One-dimensional (1D) toy model of lattice thermal transport with a local maximum temperature. (**A**) Sketch of the toy model showing the change in atomic masses. (**B**) Phonon transmission of the 1D systems (black curve) as well as Bose-Einstein distributions at the thermal reservoirs. (**C**) Local density of states along the atomic chain. (**D**) Local temperature, calculated as described in the methods section of the main manuscript. (**E**) The spectral number operator described in the main manuscript shows the number of phonons resolved in frequency.

Solution of the 2D heat equation for a delta source

Consider a two-dimensional infinite sheet with a heat source that keeps the temperature at the origin constant at T_{inj} and a heat reservoir at T_0 at the edges. The temperature profile for this system does not depend on time and has circular symmetry. Thus, the heat equation for the system reduces to

$$\frac{\partial T}{\partial t} = \alpha \nabla^2 T$$

$$\frac{\partial T}{\partial t} = \alpha \left(\frac{\partial^2 T}{\partial r^2} + \frac{1}{r}\frac{\partial T}{\partial r} + \frac{1}{r^2}\frac{\partial T}{\partial \theta} \right)$$

$$0 = \alpha \left(\frac{\partial^2 T}{\partial r^2} + \frac{1}{r}\frac{\partial T}{\partial r} \right),$$

with T the temperature, r the radial coordinate and, α the thermal diffusivity. This equation has a general solution of the form

$$T = A\mathrm{Ln}(Br),$$

with A and B constants that depend on the boundary conditions. This solution decays logarithmically with the distance from the heat source.

Temperature distribution around a ballistic source in 2D

The phonon source is a disk of radius r_0 at temperature $T + \Delta T$, where T is the background temperature (See Fig. S6). The energy density at the edge of the disk is the one corresponding to the thermal background, $E_B = \int \hbar\omega\rho(\omega) f(\omega, T) d\omega$, plus half of the extra one present in the disk, $\frac{1}{2}\Delta E_{\text{disk}} = \frac{1}{2}\Delta T \int \hbar\omega\rho(\omega)\frac{df}{dT} d\omega$. (ρ is the phonon density of states, and f is the Bose-Einstein distribution.) As you get a distance r away from the center of the disk, the disk's contribution to the local energy density goes down inversely proportional to the diameter of the circumference centered at the disk: $E(r) = E_B + \frac{r_0}{r}\frac{1}{2}\Delta E_{\text{disk}}$. On the other hand, to have the same energy density without the source, we need to increase the background temperature by δT such that $E_B(T + \delta T) = E_B(T) + \delta T \int \hbar\omega\rho(\omega)\frac{df}{dT} d\omega$. Equating $E(r) = E_B(T + \delta T)$, allows us to define the local temperature $\delta T = \frac{1}{2}\Delta T \frac{r_0}{r}$.

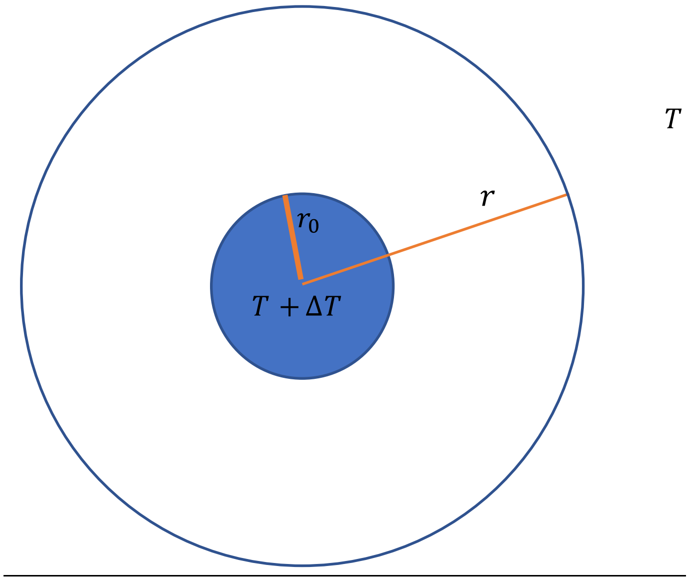

Fig. S6. Sketch of a two-dimensional ballistic source. The central blue region represents the heat source at temperate $T + \Delta T$.

Surface roughness scattering

According to Ziman (*20*), the mean free path of phonons scattering with the surfaces of a nanowire can be described as

$$\Lambda_s = \frac{1+p}{1-p}\Lambda_{Cas},$$

with Λ_{Cas} the mean free path of phonons in the purely diffusive limit and p the probability of specular reflection. Λ_{Cas} assumes that surfaces are black bodies that absorb phonons and re-emit them in all directions and is given by (*50*)

$$\Lambda_{Cas} = 1.12\sqrt{A},$$

with A the cross-sectional area. In particular, for a nanowire with $A = 0.1\mu\text{m} \times 0.1\mu\text{m}$, $\Lambda_{Cas} = 0.112\mu\text{m}$. The probability of specular reflection at a rough surface for a phonon plane wave with wavelength λ, impinging at the surface with an incident angle of θ is given by

$$p = \exp\left(-\frac{16\pi^3\eta^2}{\lambda^2}\cos^2\theta\right).$$

The roughness of the surface is described by η, the root mean square of deviation of the surface from a reference plane.

For the suspended membranes used in Tavakoli et al., where stoichiometric SiN grown by low pressure chemical vapor deposition (LPCVD) was used, the roughness is about $\eta \approx 3\text{nm}$ (*14*). Let us assume that the temperature of the system is 0.1 K. To make a back-of-the-envelope estimation of p we make $\cos^2\theta = 1$ and choose $\lambda = 0.5\mu\text{m}$, which results in $p = 0.98$ and $\Lambda_s = 12.85\mu\text{m}$. Our choice of λ corresponds to the wavelength of a phonon in the transverse acoustic branch of SiN with frequency $f = 12.25$ GHz. Thus, longitudinal and transverse acoustic phonons with a frequency less than 12.25 GHz have p larger than 0.98 and Λ_s larger than $12.85\mu\text{m}$. At 0.1 K, the integral of the mode heat capacity up to 12.25 GHz accounts for 96% of its integral over the whole frequency spectrum. Thus, at 0.1 K, most of the heat is carried by phonon with surface scattering limited mean free path larger than $12.85\mu\text{m}$.

REFERENCES AND NOTES

1. J. B. Pendry, Quantum limits to the flow of information and entropy. *J. Phys. A. Math. Gen.* **16**, 2161–2171 (1983).

2. J. P. Pekola, B. Karimi, Colloquium: Quantum heat transport in condensed matter systems. *Rev. Mod. Phys.* **93**, 041001 (2021).

3. S. Jezouin, F. D. Parmentier, A. Anthore, U. Gennser, A. Cavanna, Y. Jin, F. Pierre, Quantum limit of heat flow across a single electronic channel. *Science* **342**, 601–604 (2013).

4. N. Mosso, U. Drechsler, F. Menges, P. Nirmalraj, S. Karg, H. Riel, B. Gotsmann, Heat transport through atomic contacts. *Nat. Nanotechnol.* **12**, 430–433 (2017).

5. L. Cui, W. Jeong, S. Hur, M. Matt, J. C. Klöckner, F. Pauly, P. Nielaba, J. C. Cuevas, E. Meyhofer, P. Reddy, Quantized thermal transport in single-atom junctions. *Science* **355**, 1192–1195 (2017).

6. M. Meschke, W. Guichard, J. P. Pekola, Single-mode heat conduction by photons. *Nature* **444**, 187–190 (2006).

7. M. Partanen, K. Y. Tan, J. Govenius, R. E. Lake, M. K. Mäkelä, T. Tanttu, M. Möttönen, Quantum-limited heat conduction over macroscopic distances. *Nat. Phys.* **12**, 460–464 (2016).

8. L. G. C. Rego, G. Kirczenow, Quantized thermal conductance of dielectric quantum wires. *Phys. Rev. Lett.* **81**, 232–235 (1998).

9. K. Schwab, E. A. Henriksen, J. M. Worlock, M. L. Roukes, Measurement of the quantum of thermal conductance. *Nature* **404**, 974–977 (2000).

10. Y. Tanaka, F. Yoshida, S. Tamura, Lattice thermal conductance in nanowires at low temperatures: Breakdown and recovery of quantization. *Phys. Rev. B.* **71**, 205308 (2005).

11. C. A. Polanco, Nonequilibrium green’s functions (NEGF) in vibrational energy transport: A topical review. *Nanoscale Microscale Thermophys. Eng.* **25**, 1–24 (2021).

12. Y. Gu, J. Wang, Green's function method hybrid with the finite element method for ballistic phonon transport at low temperatures in nanostructures of arbitrary shape. *Numer. Heat Transf. Part B Fundam.* **72**, 71–81 (2017).

13. H.-Y. Chiu, V. V Deshpande, H. W. C. Postma, C. N. Lau, C. Mikó, L. Forró, M. Bockrath, Ballistic phonon thermal transport in multiwalled carbon nanotubes. *Phys. Rev. Lett.* **95**, 226101 (2005).

14. A. Tavakoli, K. Lulla, T. Crozes, N. Mingo, E. Collin, O. Bourgeois, Heat conduction measurements in ballistic 1D phonon waveguides indicate breakdown of the thermal conductance quantization. *Nat. Commun.* **9**, 4287 (2018).

15. R. Landauer, Spatial variation of currents and fields due to localized scatterers in metallic conduction. *IBM J. Res. Dev.* **1**, 223–231 (1957).

16. E. T. Swartz, R. O. Pohl, Thermal boundary resistance. *Rev. Mod. Phys.* **61**, 605–668 (1989).

17. N. Zen, T. A. Puurtinen, T. J. Isotalo, S. Chaudhuri, I. J. Maasilta, Engineering thermal conductance using a two-dimensional phononic crystal. *Nat. Commun.* **5**, 3435 (2014).

18. J. Maire, R. Anufriev, R. Yanagisawa, A. Ramiere, S. Volz, M. Nomura, Heat conduction tuning by wave nature of phonons. *Sci. Adv.* **3**, e1700027 (2017).

19. H. F. C. Hoevers, M. L. Ridder, A. Germeau, M. P. Bruijn, P. A. J. de Korte, R. J. Wiegerink, Radiative ballistic phonon transport in silicon-nitride membranes at low temperatures. *Appl. Phys. Lett.* **86**, 251903 (2005).

20. J. M. Ziman, Electrons and phonons : the theory of transport phenomena in solids (Clarendon Press, Oxford, 2001).

21. G. Srivastava, The physics of phonons (Taylor & Francis Group, New York, NY, 1990).

22. M. Asen-Palmer, K. Bartkowski, E. Gmelin, M. Cardona, A. P. Zhernov, A. V. Inyushkin, A. Taldenkov, V. I. Ozhogin, K. M. Itoh, E. E. Haller, Thermal conductivity of germanium crystals with different isotopic compositions. *Phys. Rev. B.* **56**, 9431–9447 (1997).

23. N. Mingo, L. Yang, D. Li, A. Majumdar, Predicting the thermal conductivity of Si and Ge nanowires. *Nano Lett.* **3**, 1713–1716 (2003).

24. W. Holmes, J. M. Gildemeister, P. L. Richards, V. Kotsubo, Measurements of thermal transport in low stress silicon nitride films. *Appl. Phys. Lett.* **72**, 2250–2252 (1998).

25. W. A. Phillips, Ed., Amorphous Solids: Low-Temperature Properties (Springer Berlin Heidelberg, Berlin, Heidelberg, 1981, vol. 24 of Topics in Current Physics.

26. M. C. Wingert, J. Zheng, S. Kwon, R. Chen, Thermal transport in amorphous materials: A review. *Semicond. Sci. Technol.* **31**, 113003 (2016).

27. R. C. Zeller, R. O. Pohl, Thermal conductivity and specific heat of noncrystalline solids. *Phys. Rev. B.* **4**, 2029–2041 (1971).

28. P. W. Anderson, B. I. Halperin, C. M. Varma, Anomalous low-temperature thermal properties of glasses and spin glasses. *Philos. Mag. A J. Theor. Exp. Appl. Phys.* **25**, 1–9 (1972).

29. P. B. Allen, J. L. Feldman, Thermal conductivity of disordered harmonic solids. *Phys. Rev. B.* **48**, 12581–12588 (1993).

30. C. S. O'Hern, L. E. Silbert, A. J. Liu, S. R. Nagel, Jamming at zero temperature and zero applied stress: The epitome of disorder. *Phys. Rev. E.* **68**, 011306 (2003).

31. W. Schirmacher, Thermal conductivity of glassy materials and the boson peak. *Europhys. Lett.* **73**, 892–898 (2006).

32. H. Mizuno, H. Shiba, A. Ikeda, Continuum limit of the vibrational properties of amorphous solids. *Proc. Natl. Acad. Sci. U.S.A.* **114**, E9767–E9774 (2017).

33. M. M. Leivo, J. P. Pekola, Thermal characteristics of silicon nitride membranes at sub-Kelvin temperatures. *Appl. Phys. Lett.* **72**, 1305–1307 (1998).

34. B. L. Zink, R. Pietri, F. Hellman, Thermal conductivity and specific heat of thin-film amorphous silicon. *Phys. Rev. Lett.* **96**, 55902 (2006).

35. D. R. Queen, F. Hellman, Thin film nanocalorimeter for heat capacity measurements of 30 nm films. *Rev. Sci. Instrum.* **80**, 063901 (2009).

36. D. J. Goldie, A. V Velichko, D. M. Glowacka, S. Withington, Ultra-low-noise MoCu transition edge sensors for space applications. *J. Appl. Phys.* **109**, 84507 (2011).

37. A. Tavakoli, C. Blanc, H. Ftouni, K. J. Lulla, A. D. Fefferman, E. Collin, O. Bourgeois, Universality of thermal transport in amorphous nanowires at low temperatures. *Phys. Rev. B.* **95**, 165411 (2017).

38. A. Tavakoli, K. J. Lulla, T. Puurtinen, I. Maasilta, E. Collin, L. Saminadayar, O. Bourgeois, Specific heat of thin phonon cavities at low temperature: Very high values revealed by zeptojoule calorimetry. *Phys. Rev. B.* **105**, 224313 (2022).

39. J. T. Karvonen, T. Kühn, I. J. Maasilta, Temperature profile for ballistic and diffusive phonon transport in a suspended membrane with a radial symmetric heat sources. arXiv:1008.3230 (2010). https://doi.org/10.48550/arXiv.1008.3230

40. N. Mingo, L. Yang, Phonon transport in nanowires coated with an amorphous material: An atomistic Green's function approach. *Phys. Rev. B.* **68**, 245406 (2003).

41. N. Mingo, in *Thermal Nanosystems and Nanomaterials*, S. Volz, Ed. (Springer Berlin Heidelberg, Berlin, Heidelberg, 2009; https://doi.org/10.1007/978-3-642-04258-4_3)10.1007/978-3-642-04258-4_3), pp. 63–94.

42. J.-S. Wang, J. Wang, N. Zeng, Nonequilibrium Green's function approach to mesoscopic thermal transport. *Phys. Rev. B.* **74**, 33408 (2006).

43. R. D. Cook, D. S. Malkus, M. E. Plesha, R. J. Witt, *Concepts and Applications of Finite Element Analysis* (John Wiley & Sons, Inc., ed. 4rd, 2002).

44. S. Datta, *Quantum Transport Atom to Transistor* (Cambridge University Press, New York, NY, ed. 2, 2005).

45. A. Khan, J. Philip, P. Hess, Young's modulus of silicon nitride used in scanning force microscope cantilevers. *J. Appl. Phys.* **95**, 1667–1672 (2004).

46. K. E. Petersen, Silicon as a mechanical material. *Proc. IEEE* **70**, 420–457 (1982).

47. S. Mizuno, N. Nishiguchi, Acoustic phonon modes and dispersion relations of nanowire superlattices. *J. Phys. Condens. Matter* **21**, 195303 (2009).

48. C. A. Polanco, L. Lindsay, Phonon thermal conductance across GaN-AlN interfaces from first principles. *Phys. Rev. B.* **99**, 075202 (2019).

49. C. A. Polanco, A. van Roekeghem, L. R. Lindsay, N. Mingo, fenegf repository (2022; https://bitbucket.org/capolancobb/fenegf.v0).
